# Hesitant adopters: COVID-19 vaccine hesitancy among diverse vaccinated adults in the United States

**DOI:** 10.1016/j.imj.2023.03.001

**Published:** 2023-03-25

**Authors:** Sharon Reece, Sheena CarlLee, Aaron J. Scott, Don E. Willis, Brett Rowland, Kristin Larsen, Ijanae Holman-Allgood, Pearl A. McElfish

**Affiliations:** a College of Medicine, University of Arkansas for Medical Sciences Northwest, Fayetteville, AR 72703, USA; b Office of Community Health and Research, University of Arkansas for Medical Sciences Northwest, Springdale, AR 72703, USA; c College of Medicine, University of Arkansas for Medical Sciences Northwest, Springdale, AR 72703, USA

**Keywords:** COVID-19 vaccine, Vaccine hesitancy, Hesitant adopters, US adults

## Abstract

**Background:**

Despite the United States (US) having an abundant supply of COVID-19 vaccines, vaccination rates lag behind other high-income countries, suggesting that vaccine hesitancy and attitudes play a greater role in public health measures than pure supply and access. With the acknowledgment that vaccination attitudes and status may or may not be correlated, this study examined COVID-19 vaccine hesitancy among vaccinated US adults by asking: 1) What is the prevalence of COVID-19 vaccine hesitancy among the vaccinated? 2) Does COVID-19 vaccine hesitancy vary across sociodemographic characteristics? 3) Does COVID-19 vaccine hesitancy vary by healthcare access and influenza vaccination over the past 5 years?

**Methods:**

Data were collected through an online survey of 2022 US adults with a final analytic sample of 1383 vaccinated respondents.

**Results:**

Overall, 48.8% of vaccinated adults reported some level of hesitancy, while a slight majority reported they were “not at all hesitant”. Younger respondents, women, and Black and American Indian or Alaska Native participants had greater adjusted odds of being more hesitant towards receiving the COVID-19 vaccine. Respondents who had a primary care physician had greater adjusted odds than those who did not have a primary care physician of being more hesitant towards receiving the COVID-19 vaccine.

**Conclusions:**

This is the first population-based national sample study examining COVID-19 vaccine hesitancy among vaccinated individuals from subgroups of distinctive backgrounds in order to inform targeted strategies for reducing vaccine hesitancy. Findings can assist in efforts to increase vaccination rates and also decrease vaccine hesitancy at the national level.

## 1 Introduction

The World Health Organization has identified vaccine hesitancy as a global health concern, and while it is not a new phenomenon, vaccine hesitancy has been amplified during the COVID-19 pandemic [1]. Despite the United States (US) having an abundant supply of COVID-19 vaccine doses [2], the vaccination rate lags behind other high-income countries [3], suggesting that vaccine hesitancy and attitudes toward vaccination play a greater role in public health measures than pure supply and access [4]. Prior research suggests that vaccine hesitancy was greater among women, minority, non-metro/rural residents, those who have foregone medical care due to cost, and those receiving fewer influenza vaccinations over the past 5 years [5], [6], [7], [8], [9], [10].

Vaccine hesitancy refers to a wide-ranging continuum of attitudes that are important for understanding vaccine uptake but do not overlap perfectly with vaccination behaviors [11,12]. Yet, vaccine hesitancy and vaccination status are often conflated. Studies of vaccine hesitancy published in highly reputable medical journals have conflated vaccination status and the attitude of hesitancy [13]. Emerging research demonstrates individuals who have been vaccinated commonly report some degree of vaccine hesitancy either months before receiving the vaccine, or even the same day as having received it [14], [15], [16], [17], [18], [19], [20], [21]. Therefore, this study distinguishes vaccine hesitancy (an attitude) from vaccination status (a behavior) [22] and recognizes individuals who are “hesitant adopters” (*ie*, individuals who were both hesitant and vaccinated). Prior research on hesitant adopters has focused on small samples [21,23], qualitative analysis [24], and single state geographic samples [19]. Given the past research demonstrating how common vaccine hesitancy is among the vaccinated [14], [15], [16], [17], [18], [19], [20], [21], it is important to consider the presence of vaccine hesitancy and its correlates even among individuals who have received a dose of the COVID-19 vaccine. To our knowledge, there are no studies examining hesitancy among vaccinated individuals in a diverse sample of US adults.

The purpose of this study is to examine COVID-19 vaccine hesitancy among vaccinated US adults. A more nuanced understanding of hesitancy among vaccinated individuals at the national level is critical to increase vaccine uptake. Moreover, since vaccination sequences often require more than one dose, it is important not to ignore the presence and correlates of hesitancy among those who have already received at least one dose. The vaccinated represent a group who are known to be open-to a degree-to vaccination. Understanding correlates of their vaccine hesitancy is critical to vaccine uptake because their continued vaccination compliance is not guaranteed. Further, understanding correlates of hesitancy among the vaccinated can provide insights into what factors might need to be addressed to maintain their compliance for the full sequence of doses. With the acknowledgment that vaccine attitudes and vaccination status may or may not be correlated [11], the research questions are: 1) What is the prevalence of COVID-19 vaccine hesitancy among the vaccinated? 2) Does COVID-19 vaccine hesitancy vary across sociodemographic characteristics? 3) Does COVID-19 vaccine hesitancy vary by healthcare access and influenza vaccination over the past 5 years?

## 2 Materials and methods

### 2.1 Procedures

The sampling frame consisted of an online opt-in panel of research volunteers across the United States established by Atomik Research. Respondents completed the survey via the online survey platform Decipher. Recruitment occurred between September 7, 2021 and October 3, 2021. English and Spanish versions of the survey were available to respondents. Inclusion criteria required respondents be 18 years of age or older and reside in the United States in order to take part in the survey. Recruitment involved informing potential respondents about: 1) the estimated duration (10 minutes), 2) potential risks and benefits, 3) the voluntary nature of participation, and 4) confidentiality of responses. Consent to participate was indicated by agreeing to complete the survey. Study procedures were reviewed and approved by the University of Arkansas for Medical Sciences Institutional Review Board (#263020).

**Fig. 1:**
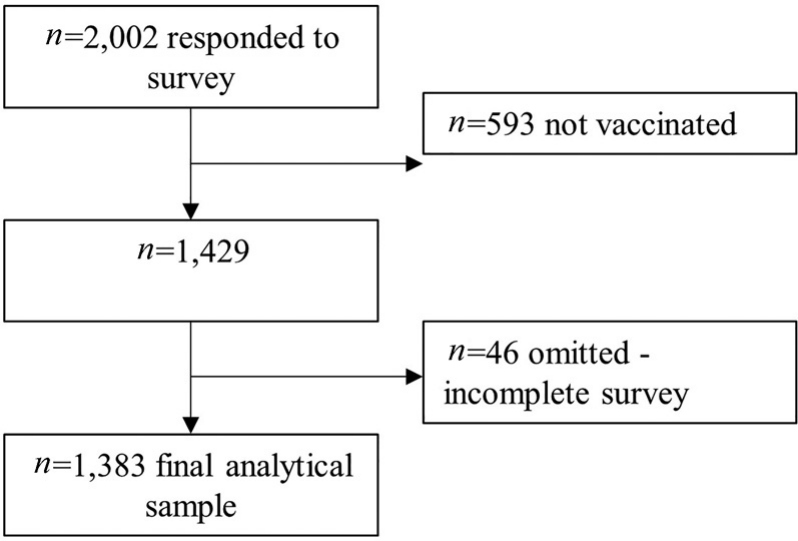
Participant flow.

A total of 2022 adults responded to the survey. Selection criteria were applied to the sample in 2 stages in order to answer the research question. First, the sample was limited to those who reported they had been vaccinated against COVID-19 with at least 1 dose of a COVID-19 vaccine (*n* = 1429; 71.4%). Second, ordinal regression requires complete cases for analysis. Of the 1,429 who reported a prior COVID-19 vaccination, those with incomplete responses to other items included in the model were omitted from analyses (*n* = 46; 3.2%). This provided a final analytical sample of 1,383 vaccinated respondents (Fig. 1). Our analysis included respondents from 49 US states, 2 US territories, and the District of Columbia.

Minority populations, including Asian, Black/African American, Hispanic/Latinx, American Indian or Alaska Native (AIAN), and Native Hawaiian or Pacific Islander (NHPI) individuals, were oversampled in order to avoid aggregation of racial and ethnic group data. The oversampling strategy resulted in an overall, unweighted sample comprised of 15% Asian, 20% Black/African American, 20% Hispanic/Latinx, 13% AIAN, 12% NHPI, and 20% White respondents. Inadequate sampling of these groups can mask differences in lived experiences, health behaviors, and attitudes [25,26]. Data were weighted using the random iterative method [27]. This method ensures data are representative of the general US adult population across demographic variables: gender (men, women, non-binary); race and ethnicity (Asian, Black/African American, Hispanic/Latinx, AIAN, NHPI, and White); and age (18–24, 25–34, 35–44, 45–4, 55–64, 65+).

### 2.2 Measures

#### 2.2.1 Vaccine hesitancy

The dependent variable was an ordinal measure of COVID-19 vaccine hesitancy. Vaccinated respondents were asked, “Thinking specifically about the COVID-19 vaccines, how hesitant were you about getting vaccinated?” Response options included “not at all hesitant” “a little hesitant” “somewhat hesitant” and “very hesitant”.

#### 2.2.2 Sociodemographic characteristics

Age was calculated from respondents’ reported year of birth and was categorized into 4 groups: 18–29, 30–44, 45–59, 60+. Gender was reported as man or woman. Very few individuals (*n* = 8) reported a gender other than man or woman. Race/ethnicity included 6 categories: non-Hispanic White, non-Hispanic Black/African American, Hispanic/Latinx (of any race), non-Hispanic Asian, AIAN, and NHPI. Participants reported their highest level of education completed. Responses were grouped into: high school degree/graduate equivalency degree or lower, some college/associate degree, and bachelor's degree or higher. Marital status was grouped into: married/coupled (married or member of an unmarried couple) and unmarried/single (divorced, widowed, separated, or never married).

#### 2.2.3 Healthcare access

Healthcare access was measured across 5 items: 1) “Do you have any kind of healthcare coverage, including health insurance, prepaid plans, such as HMOs, government plans, such as Medicare, or Indian Health Service?” (yes, no); 2) “Do you have one person you think of as your personal doctor or healthcare provider?” (yes, no); 3) “Was there a time in the past 12 months when you needed a doctor but could not see one because of the cost?” (yes, no); 4) “About how long has it been since you last visited a doctor for a routine checkup?” (in the past year, in the past 2 years, in the past 5 years, 5 or more years ago, never). Due to changes in healthcare seeking behaviors as a result of the COVID-19 pandemic, these responses were grouped into 2 categories: in the past 2 years and more than 2 years ago; and 5) “How many years in the past 5 years have you gotten a seasonal flu vaccine?” (never, 1–2 years, 3–4 years, every year).

### 2.3 Statistical analyses

Data were managed, cleaned, and analyzed using SAS 9.4. Weighted descriptive statistics are presented by level of vaccine hesitancy. A weighted multivariable ordinal logistic regression was used to examine correlates of vaccine hesitancy, controlling for the sociodemographic and healthcare access variables outlined above. The Brant test indicated the assumption of proportional odds was met (*p* = 0.3660). All independent variables were set to use a reference group that produced adjusted odds ratios above 1.00 for ease of interpretation.

## 3 Results

### 3.1 Descriptives

Table 1 provides weighted percentages among respondents vaccinated against COVID-19. Approximately one-third (36.2%) of respondents were 60 years of age or older. Approximately half (51.2%) of respondents were women, and less than half (43.7%) were White. Less than one-quarter (22.5%) reported their highest level of education as a high school degree or lower, and approximately half (50.9%) were married or in a coupled relationship.

**Table 1:** Sociodemographic, healthcare access, vaccine behaviors and hesitancy among participants vaccinated for COVID-19 (*n* = 1,383).

	Not at all hesitant	A little hesitant	Somewhat hesitant	Very hesitant	Total
Measures	%	%	%	%	%
Age group					
18–29	11.5	15.5	17.2	22.1	14.3
30–44	18.4	27.4	30.0	38.9	24.2
45–59	24.4	29.6	24.1	19.1	25.3
60+	45.6	27.5	28.7	19.8	36.2
*Gender*					
Men	53.7	46.3	38.3	43.2	48.8
Women	46.3	53.7	61.7	56.8	51.2
Race/ethnicity					
White	47.5	44.0	38.0	29.6	43.7
Black/African American	14.1	18.0	20.9	29.6	17.5
Hispanic/Latinx	18.5	17.3	20.5	25.1	19.1
Asian	13.9	12.8	10.7	5.8	12.5
American Indian or Alaska Native	2.2	3.4	5.4	4.9	3.2
Native Hawaiian or Pacific Islander	3.7	4.5	4.5	5.1	4.1
Education					
HS degree/GED or lower	21.4	22.8	24.6	24.9	22.5
Some college/associate degree	31.8	33.1	37.4	38.6	33.5
Bachelor's degree or higher	46.8	44.2	38.0	36.5	44.1
Marital status					
Married/coupled	50.9	50.4	51.8	51.6	50.9
Unmarried/single	49.1	49.6	48.2	48.4	49.1
Healthcare coverage					
Yes	93.2	88.1	91.0	80.5	90.3
No	6.8	12.0	9.0	19.5	9.7
Primary care physician					
Yes	88.3	84.7	89.4	84.2	87.1
No	11.7	15.3	10.7	15.8	12.9
No medical care due to cost					
Yes	10.6	18.3	20.1	25.2	15.3
No	89.4	81.7	79.9	74.8	84.7
Routine doctor checkup					
In the past 2 years or less	89.8	85.8	86.9	81.0	87.5
More than 2 years ago	10.2	14.2	13.1	19.0	12.5
5-year influenza vaccination					
Never	14.2	28.1	19.5	29.6	20.1
1–2 years	18.9	22.7	26.0	27.9	21.6
3–4 years	9.6	12.8	11.8	11.0	10.8
Every year	57.4	36.5	42.8	31.5	47.4
COVID-19 vaccine hesitancy					
Not at all hesitant	–	–	–	–	51.2
A little hesitant	–	–	–	–	27.5
Somewhat hesitant	–	–	–	–	11.7
Very hesitant	–	–	–	–	9.6

All percentages are weighted.

Percentages may not total 100% due to rounding.

HS, high school; GED, graduate equivalency degree.

Among healthcare access variables, the majority had healthcare coverage (90.3%), a primary care physician (87.1%), did not have to forego medical care due to cost (84.7%), and had a routine doctor checkup in the past 2 years or less (87.5%). Approximately half (47.4%) reported they were vaccinated against influenza every year over the past 5 years, and approximately half (48.8%) reported some degree of hesitancy toward getting the COVID-19 vaccine.

### 3.2 Weighted ordinal logistic regression

Younger respondent groups (18–29 and 30–44) had 2.46 and 2.77 times greater adjusted odds, respectively, of being more hesitant toward receiving the COVID-19 vaccine than the oldest participants (60+). Women had 1.67 times greater adjusted odds than men of being more COVID-19 vaccine hesitant. Black/African American and AIAN participants had 1.71 and 1.76 times greater adjusted odds, respectively, than White participants of being more hesitant toward receiving the COVID-19 vaccine. Married/coupled respondents had 1.28 times greater adjusted odds than unmarried/single participants of being more COVID-19 vaccine hesitant (Table 2).

**Table 2:** Weighted ordinal logistic regression-COVID-19 vaccine hesitancy (*n* = 1,383).

	B	SE	*p*	aOR (95%CI)
Age group				
18–29	0.90	0.182	**<0.001**	2.46 (1.72, 3.52)
30–44	1.02	0.148	**<0.001**	2.77 (2.08, 3.71)
45–59	0.53	0.142	<**0.001**	1.69 (1.28, 2.24)
60+	–	–	–	–
Gender				
Women	0.51	0.106	**<0.001**	1.67 (1.36, 2.06)
Men	–	–	**–**	–
Race/ethnicity				
Black/African American	0.54	0.145	**<0.001**	1.71 (1.29, 2.27)
Hispanic/Latinx	0.07	0.148	0.660	1.07 (0.80, 1.43)
Asian	−0.18	0.176	0.311	0.83 (0.59, 1.18)
American Indian or Alaska Native	0.56	0.288	**0.0497**	1.76 (1.00, 3.09)
Native Hawaiian or Pacific Islander	0.01	0.266	0.962	1.01 (0.60, 1.71)
White	–	–	–	–
Education				
HS degree/GED or lower	0.19	0.139	0.164	1.21 (0.92, 1.59)
Some college/associate degree	0.22	0.122	0.068	1.25 (0.98, 1.59)
Bachelor's degree or higher	–	–	–	–
Marital status				
Married/coupled	0.25	0.108	**0.023**	1.28 (1.04, 1.58)
Unmarried/single	–	–	–	–
Healthcare coverage				
No	0.30	0.180	0.096	1.35 (0.95, 1.92)
Yes	–	–	–	–
Primary care physician				
Yes	0.46	0.179	**0.010**	1.59 (1.12, 2.25)
No	–	–	**–**	–
No medical care due to cost				
Yes	0.41	0.144	**0.005**	1.50 (1.13, 1.99)
No	–	–	–	–
Routine doctor checkup				
More than 2 years ago	0.33	0.172	0.052	1.53 (0.91, 2.56)
In the past 2 years or less	–	–	**–**	–
5-year influenza vaccination				
Never	0.67	0.141	**<0.001**	1.95 (1.48, 2.57)
1–2 years	0.37	0.139	**0.007**	1.45 (1.11, 1.91)
3–4 years	0.36	0.173	**0.037**	1.44 (1.02, 2.02)
Every year	–	–	**–**	–

Bold *p*-values represent statistical significance based on an alpha of 0.05.

HS, high school; GED, graduate equivalency degree; aOR, adjusted odds ratio.

Respondents who had a primary care physician had 1.59 times greater adjusted odds than those who did not have a primary care physician of being more hesitant toward receiving the COVID-19 vaccine. Respondents who had to forego medical care due to cost in the past year had 1.50 times greater adjusted odds of being more COVID-19 vaccine hesitant than those who did not have to forego medical care due to cost. Respondents who had received the flu vaccine never, 1–2 years, and 3–4 years out of the past 5 years had 1.95, 1.45, and 1.44 times greater adjusted odds, respectively, of being more hesitant toward receiving the COVID-19 vaccine than participants who received the flu vaccine every year (Table 2).

## 4 Discussion

This study examined hesitancy among those who received the COVID-19 vaccine. The analysis included descriptive statistics and a weighted multivariable ordinal logistic regression to examine correlates of vaccine hesitancy among 1,383 vaccinated participants. Overall, 48.8% of those who received a COVID-19 vaccine reported some level of hesitancy, while a slight majority reported they were “not at all hesitant.” Younger respondents had greater adjusted odds than the oldest participants of being more hesitant toward receiving the COVID-19 vaccine, which is consistent with existing literature [23]. Women had greater adjusted odds than men of being more COVID-19 vaccine hesitant, which is also consistent with prior literature [14,^28^,29]. Black and AIAN participants had greater adjusted odds of being more hesitant towards receiving the COVID-19 vaccine. The finding of more hesitancy among Black/African American individuals is consistent with other literature [14]. The finding of more hesitancy among AIAN individuals is also consistent with other literature finding AIAN individuals had the lowest trust in the scientific community compared with White, Black, and Asian individuals [30]. While we examined race/ethnicity as a predictor, it is likely historical trauma and historic and contemporary racism and medical mistreatment of AIAN and Black/African communities has perpetuated greater hesitancy [31], [32], [33], [34], [35], [36], [37]. As healthcare providers seek to improve vaccination rates, it is important to understand that many minoritized communities may choose to be vaccinated while also maintaining a higher level of hesitancy.

Respondents who had a primary care physician had greater adjusted odds than those who did not have a primary care physician of being more hesitant toward receiving the COVID-19 vaccine. Prior studies have shown a positive correlation between number of primary care physicians per capita with a higher rate of COVID-19 vaccination [38]. This unexpected finding could represent a phenomenon where a patient's behavior, but not attitude, is influenced by their primary care physician. This finding emphasizes the importance of distinguishing vaccine hesitancy from vaccine status.

### 4.1 Strengths and limitations

This study included a large number of responses from a diverse sample of adults throughout the United States with disaggregated sampling from Asian, AIAN, and NHPI individuals. Avoiding aggregation allows for analysis that recognizes the heterogeneity and diversity of lived experiences between distinctive racial/ethnic groups. The limitations surrounding methodology include recall bias such that respondents may be selective or inaccurate with their self-reported answers. This study is also limited by potential social desirability bias such that respondents may report what they view as more socially acceptable answers to the surveyors. Despite the use of a large nation-wide sample and weighting of the data, it is unlikely the nonrandom sample from which the data were collected is representative of the national population. Future research should consider mediation analysis to provide more insight into the mechanisms leading to higher vaccine hesitancy among some vaccinated populations. Exploring such mechanisms could paint a clearer picture of the processes which produce or reproduce vaccine hesitancy and, in turn, a better understanding of where interventions might be most successful. This study was unable to differentiate between individuals who had received one or multiple doses who might have differing correlates of vaccine hesitancy. The study examined self-reported prior hesitancy at the time of vaccination and did not examine further hesitancy, which needs further examination in future research. Finally, this study is unable to conclude causation, only correlation between reported themes and degrees of vaccine hesitancy.

## 5 Conclusions

This is the first population-based national sample study reporting on diverse hesitant adopters in the United States with disaggregation of groups, which are often aggregated together, obscuring any potential differences. Examination of hesitant adaptors provides new insight for healthcare providers, regulatory bodies, and policy makers. In the effort to improve vaccination rates, it is important to consider that many minoritized communities may still get vaccinated despite having high levels of hesitancy. Despite its limitations, the findings in this study can assist in efforts to increase vaccination rates and also decrease vaccine hesitancy at the national level.

## Funding

This research is supported by the University of Arkansas for Medical Sciences Translational Research Institute funding awarded through the Center for Advancing Translational Sciences of the National Institutes of Health (NIH) (UL1 TR003107); Rapid Acceleration of Diagnostics (RADx) (NIH 3 R01MD013852-03S2); and Community Engagement Alliance (CEAL) Against COVID-19 Disparities (NIH 10T2HL156812-01). This research and publication were made possible in part because of the Arkansas IDeA Network of Biomedical Research Excellence (INBRE) program funded by the National Institute of General Medical Sciences (NIGMS) (P20 GM103429) from the NIH. This research was also supported by the Health Resources and Services Administration (HRSA) of the U.S. Department of Health and Human Services (HHS) (6 U3UHS45467‐01‐01). The contents are those of the author(s) and do not necessarily represent the official views of, nor an endorsement by, HRSA, HHS, or the U.S. Government. For more information, please visit HRSA.gov. The funders of this research and publication had no role in study design, data collection and analysis, decision to publish, or preparation of the manuscript.

## Author contributions

S.R.: Writing – original draft. S.C: Writing – review & editing. A.J.S. Formal analysis. D.E.W.: Formal analysis, Writing – review & editing. B.R.: Formal analysis. K.L.: Writing – review & editing. I.H-A.: Writing – review & editing. P.A.M.: Funding acquisition, Writing – original draft, Conceptualization.

## Acknowledgments

None.

## Declaration of competing interest

Dr. Sheena CarlLee reports owning some Pfizer stock. All other authors reported no conflicts of interest.

## Data available statement

The deidentified data underlying the results presented in this study may be made available upon reasonable request from the corresponding author, Dr. Pearl A. McElfish, at pamcelfish@uams.edu.

## Ethics statement

Study procedures were reviewed and approved by the University of Arkansas for Medical Sciences Institutional Review Board (#263020).

## Informed consent

Consent to participate was indicated by agreeing to complete the survey.
